# Clinic and genetic similarity assessments of atypical carcinoid, neuroendocrine neoplasm with atypical carcinoid morphology and elevated mitotic count and large cell neuroendocrine carcinoma

**DOI:** 10.1186/s12885-022-09391-w

**Published:** 2022-03-24

**Authors:** Ying Zhang, Weiya Wang, Qianrong Hu, Zuoyu Liang, Ping Zhou, Yuan Tang, Lili Jiang

**Affiliations:** 1 Department of Pathology, West China Hospital, Sichuan University, Sichuan Province, Guoxuexiang 37, Chengdu, 610041 China; 2 West China School of Medicine, Sichuan University, Sichuan Province, Chengdu, 610041 China

**Keywords:** Atypical carcinoid morphology, Elevated mitotic count, Atypical carcinoid, Large cell neuroendocrine carcinoma

## Abstract

**Background:**

Pulmonary neuroendocrine neoplasms can be divided into typical carcinoid, atypical carcinoid, large cell neuroendocrine carcinoma, and small cell (lung) carcinoma. According to the World Health Organization, these four neoplasms have different characteristics and morphological traits, mitotic counts, and necrotic status. Importantly, “a grey-zone” neoplasm with an atypical carcinoid-like morphology, where the mitotic rate exceeds the criterion of 10 mitoses per 2 mm^2^, have still not been well classified. In clinical practice, the most controversial area is the limit of 11 mitoses to distinguish between atypical carcinoids and large cell neuroendocrine carcinomas.

**Methods:**

Basic and clinical information was obtained from patient medical records. A series of grey-zone patients (*n* = 8) were selected for exploring their clinicopathological features. In addition, patients with atypical carcinoids (*n* = 9) and classical large cell neuroendocrine carcinomas (*n* = 14) were also included to compare their similarity to these neoplasms with respect to tumour morphology and immunohistochemical staining.

**Results:**

We found that these grey-zone tumour sizes varied and affected mainly middle-aged and older men who smoked. Furthermore, similar gene mutations were found in the grey-zone neoplasms and large cell neuroendocrine carcinomas, for the mutated genes of these two are mainly involved in PI3K-Akt signal pathways and Pathways in cancer, including a biallelic alteration of *TP53/RB1* and *KEAP1*.

**Conclusions:**

Our findings indicate that neuroendocrine neoplasm with atypical carcinoid morphology and elevated mitotic counts is more similar to large cell neuroendocrine carcinoma than atypical carcinoid. Furthermore, this study may help improve diagnosing these special cases in clinical practice to avoid misdiagnosis.

**Supplementary Information:**

The online version contains supplementary material available at 10.1186/s12885-022-09391-w.

## Background

The World Health Organization (WHO) has added large cell neuroendocrine carcinoma (LCNEC) to the classification of pulmonary neuroendocrine neoplasms (pNENs) for the first time [1]. The 2017 consensus conference of the International Agency for Research on Cancer suggests that neuroendocrine neoplasms (NENs) are subdivided into well-differentiated neuroendocrine tumours (NETs) and poorly differentiated neuroendocrine carcinomas (NECs) [2–12]. Thus, in their newest edition, WHO divides pNENs into two groups (i) NETs, comprising typical carcinoid (TC) and atypical carcinoid (AC); (ii) and NECs, comprising LCNEC and small cell (lung) carcinoma (SCLC) [13].

NENs are relatively rare, and 20–30% develop in the lung [14]. Within the lung, 95% of NENs are NECs, with NETs accounting for only a small proportion [13]. The diagnostic criteria of pNENs are clearly defined based on their morphological traits, mitotic counts, and necrotic status [13]. Moreover, NETs characteristically do not occur in combination with LCNEC or SCLC [13], and differences are indeed exhibited in the biological behaviour, therapeutic consideration, the clinical prognosis of NET and NEC [5, 15, 16].

However, a grey-zone does exist as some pNENs have an AC-like morphology with elevated mitotic counts over 10 per 2 mm^2^ (AC-h), although they have only been investigated in a few studies [8, 17–19]. Due to their rare prevalence and the lack of specific classification, it is still difficult to characterise these tumours. Therefore, updates should be made to the existing classification system. To that end, additional studies to classify the characteristics of AC-h need to be carried out. As such, we conducted this retrospective study to explore the similarities and differences among AC-h, AC, and LCNEC.

## Methods

### Sample selection

Forty-four samples of surgical resected primary untreated ACs and LCNECs diagnosed between January 1, 2016 and January 1, 2021 were collected from the specimen bank of the Department of Pathology, West China Hospital, Sichuan University, with the approval of the Institutional Ethics Committee (NO: 2020 (120)). All specimens were reviewed by two experienced pathologists, and a multi-head microscope was used for joint judgment with the participation of a third professional pathologist if the results were inconsistent, based on the new 5^th^ edition WHO.

After reviewing all the slides of 44, eight AC-h were selected, meanwhile, considering the preservation time of the wax block, this study only included AC samples after January 1, 2018. Finally, 31 samples, consisting of nigh ACs, eight AC-hs and 14 LCNECs (the data had been previously collected which could be found in doi: 10.1186/s13000-022-01204-9.), were enrolled from 31 independent patients. Overall survival (OS), identified from the resection date to the cutoff date of follow-up (June 1, 2021), was identified as the primary survival outcome in this study, due to case 25 whose tumour could not be completely removed.

### Immunohistochemical analysis

Antibodies against CD56 (clone UMAB83 and BIO), synaptophysin (Syn, polyclonal, MXB), chromogranin A (CgA, clone EP38, and BIO), TTF-1 (clone 8G7G3/1, and ZECA), and Ki67 (clone MIB-1) were used for immunohistochemical (IHC) staining of all samples. Blinded to all patients’ information, two experienced pathologists assessed IHC expression independently. Controversial cases were revaluated under a multi-head microscope for joint judgment with the participation of a third professional respiratory diagnostic pathologist.

### DNA extraction and next-generation sequencing

According to the manufacturer’s instructions, DNA was extracted by a QIAamp DNA FFPE Tissue Kit (Qiagen, Carlsbad, CA, USA) after twice of de-paraffinized by xylene. Extracted DNA was purified and qualified employing the Nanodrop2000 (Thermo), and then using Qubit3.0 (Life Technology) with a dsDNA HS Assay Kit (Life Technology) to quantify DNA.

Amplified and purified DNA Libraries by PCR and then pooled together 1-2 μg of different libraries for targeted enrichment. Hybridization-based target enrichment was carried out with NimbleGen SeqCap EZ Hybridization and Wash Kit (Roche). Captured libraries by Dynabeads M-270 (Life Technologies) were amplified in KAPA HiFi HotStart ReadyMix (KAPA Biosystems), followed by purification by Agencourt AMPure XP beads. Customized xGen lockdown probes panel (Integrated DNA Technologies) were used to targeted enrich for 425 predefined genes. The enriched libraries were sequenced on Hiseq 4000 NGS platforms (Illumina) to coverage depths of at least 100 × and 300 × after removing PCR duplicates for tumour and normal tissue, respectively.

### Bioinformatics analysis

Base calling analysis was used to transfer original image data into raw sequence data, which contained sequence information and corresponding sequencing quality information. Single nucleotide variants (SNVs) and short insertions or deletions (indels) were identified by VarScan2. In-house-developed software was used to detect Copy number variations (CNVs).

### Statistical analysis

Statistical Package for the Social Sciences version 25.0 statistical software (SPSS Inc., Chicago, IL, USA) and the Kyoto Encyclopaedia of Genes and Genomes website (KEGG web) were used to conduct statistical analysis and query the gene mutation pathways, respectively. Continuous data were evaluated by were assessed by one-way analysis of variance (ANOVA). Categorical data were assessed by Pearson’s chi-squared test or Fisher’s exact test. The Kaplan–Meier method was used for survival analysis. *P* < 0.05 was considered statistically significant.

## Results

### Clinical information

**Fig. 1:**
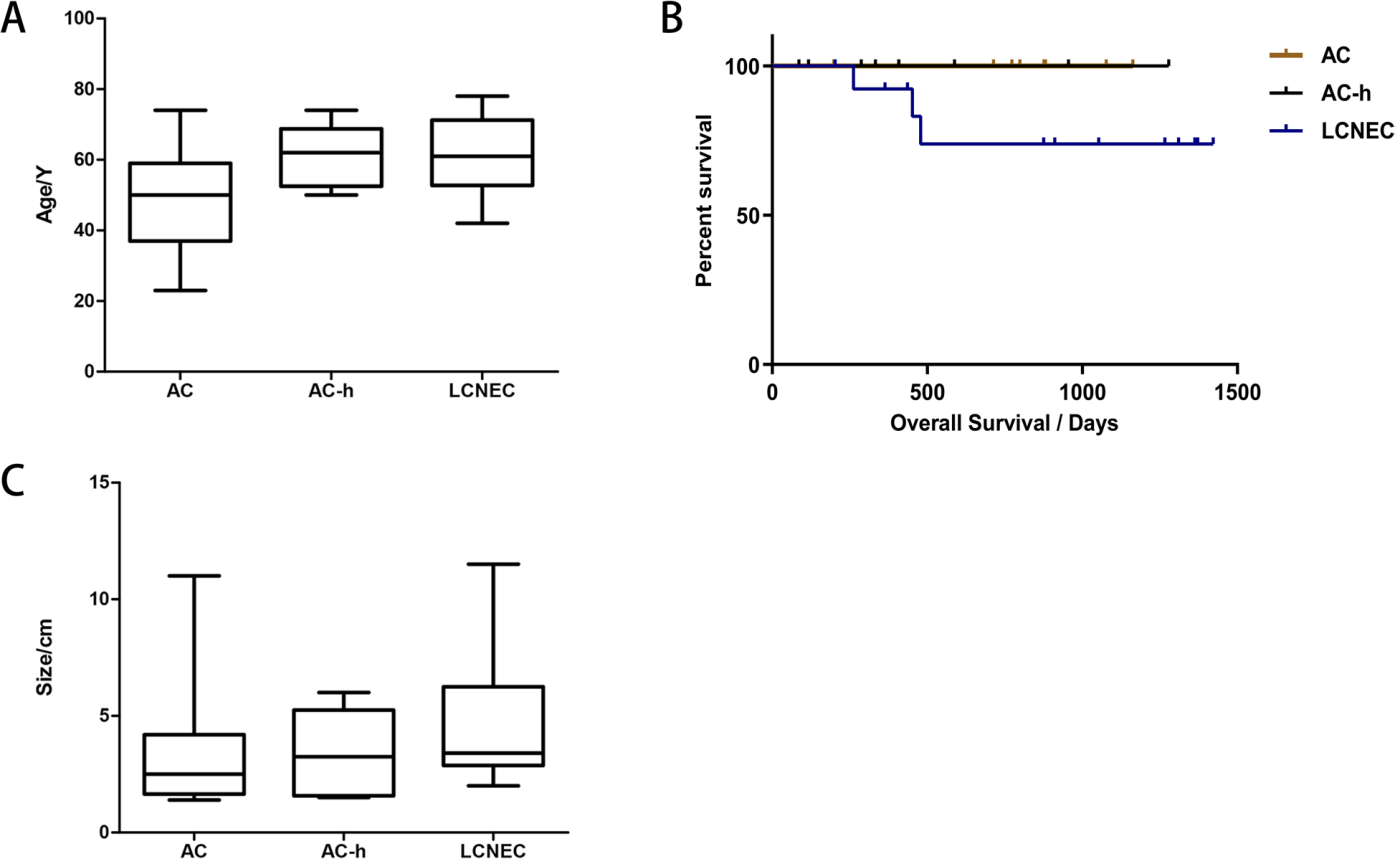
Abbreviations: *AC* Atypical carcinoid, *AC-h* Atypical carcinoid morphology with increased mitotic counts, *LCNEC* Large cell neuroendocrine carcinoid, **A**) the age-specific box diagram of the three groups of cases; **B**) the overall survival in 31 patients with AC, AC-h and LCNEC; **C**) the tumour size-specific box diagram of the three groups of cases

Basic information of the patients in the cohort is presented in Table 1 and Fig. 1A. There was a significant difference in average age at diagnosis between the three groups (*P* = 0.048) and smoking status (*P* = 0.028). In all 31 patients, asymptomatic patients were most commonly seen in the AC group. More than half the patients with AC-h or LCNEC were symptomatic; coughing was the most common symptom, followed by expectoration. In 77.8% of patients, the ACs were clinically staged I or II, far greater than that of the other two groups of tumours (Table 2). Moreover, the follow-up analyses of 28 patients showed no recurrence, metastasis, or death among the AC group (Fig. 1B). Patient’s postoperative treatment and prognosis are shown in Table 3.

**Table 1:** The demographic characteristics and smoking status of 31 samples

Characeristics	AC	AC-h	LCNEC	*P*-value
Age (years				**0.048**
< 40	**2**	**0**	**0**	
40–49	**2**	**0**	**1**	
50–59	**3**	**3**	**6**	
60–69	**1**	**4**	**3**	
> 70	**1**	**1**	**4**	
Range	**23–74**	**50–74**	**42–78**	
Mean	**49**	**61**	**61**	
M:F	**5:4**	**7:1**	**13:1**	**0.074**
Smoking				**0.028**
Never	**6**	**2**	**2**	
Has/Had	**3**	**6**	**12**	

*Abbreviations:* *AC* Atypical carcinoid, *AC-h* Atypical carcinoid morphology with increased mitotic counts, *LCNEC* Large cell neuroendocrine carcinoid, *P*-value The associations of age was assessed by One-Way ANOVA, meanwhile, other information were assessed by Pearson’s chi-squared test or Fisher’s exact test and *P* < 0.05 was considered statistically significant for all results; Age: at diagnosed; Range: the range of diagnosed age; M:F: male: female; Smoking: smoking status; Never: never smoker; Has/Had: still smoking/previous smoker

**Table 2:** The clinical information and preoperative imaging data of 31 samples

Variable	AC	AC-h	LCNEC	*P*-value
Lung lobe				
Left lung	5 (55.6%)	4 (50.0%)	6 (42.9%)	0.833
Right lung	4 (44.4%)	4 (50.0%)	8 (57.1%)	
Upper lobe	2	6	7	0.030
Others	7	2	7	
Type				0.183
Central	3 (33.3%)	2 (25.0%)	4 (28.6%)	
Peripheral	6 (66.7%)	4 (50.0%)	10 (71.4%)	
Unknown	0	2	0	
Tumor size (cm)				0.503
≤ 5	8	5	10	
> 5	1	1	4	
Unknown	0	2	0	
Stage				0.056
I,II	7	2	7	
III,IV	2	4	7	
Unknown	0	2	0	
Symptom				
Asymptomatic	6 (66.7%)	3 (37.5%)	6 (42.9%)	
Cough	2	3	7	
Expectoration	2	2	5	
Hemoptysis	0	1	2	
Chest pain	0	0	3	
Expiratory dyspnea	1	2	0	

*Abbreviations:* *Central* Central type of lung cancer, *Peripheral* Peripheral type of lung cancer, *Tumour* size The value took from the surgical records, *Stage* Evaluated basing on the Eighth Edition of the American Joint Committee on Cancer (AJCC) guidelines, *Symptom* When they first found the mass on lung, *Asymptomatic* Asymptomatic cases, for the size of operation of case 8 was not queried, the value from preoperative imaging was took to indicate the size; the patient of umber 12 who underwent lung transplantation due to severe chronic obstructive pulmonary disease and pathological examination of the diseased lung showed tumours, but no tumour evidence was found in preoperative imaging thus the tumour location, tumour size and stage could not be judged; *P*-value: the associations of tumour size was assessed by One-Way ANOVA, meanwhile, other information were assessed by Pearson’s chi-squared test or Fisher’s exact test and 0.05 was considered as statistically significant results

**Table 3:** The postoperative treatment and prognosis of 31 samples

Group	Samples	Postoperative treatment				Prognosis		
		A	B	C	D	Loss	Death	Alive
AC	9				9	0	0	9
AC-h	8				6	2	2*	4
LCNEC	14	5	1	2	5	1	4	9

*Abbreviations:* *Loss* The contact information left was empty or out of service, *Death* Died of tumour recurrence or metastasis, *: death after lung transplantation

### Imaging data

Preoperative chest CT scans were reviewed to determine tumour location (Table 2). Tumours involving the carina or a main segmental bronchus were defined as central, while the others were defined as peripheral. The primary tumour mass occurred preferentially in the periphery in these three groups. In addition, AC-h showed a clearer tendency than AC to occur in the upper lobe (*P* = 0.030) and a more stable range of tumour size fluctuations (Fig. 1C).

### Pathological findings

**Fig. 2:**
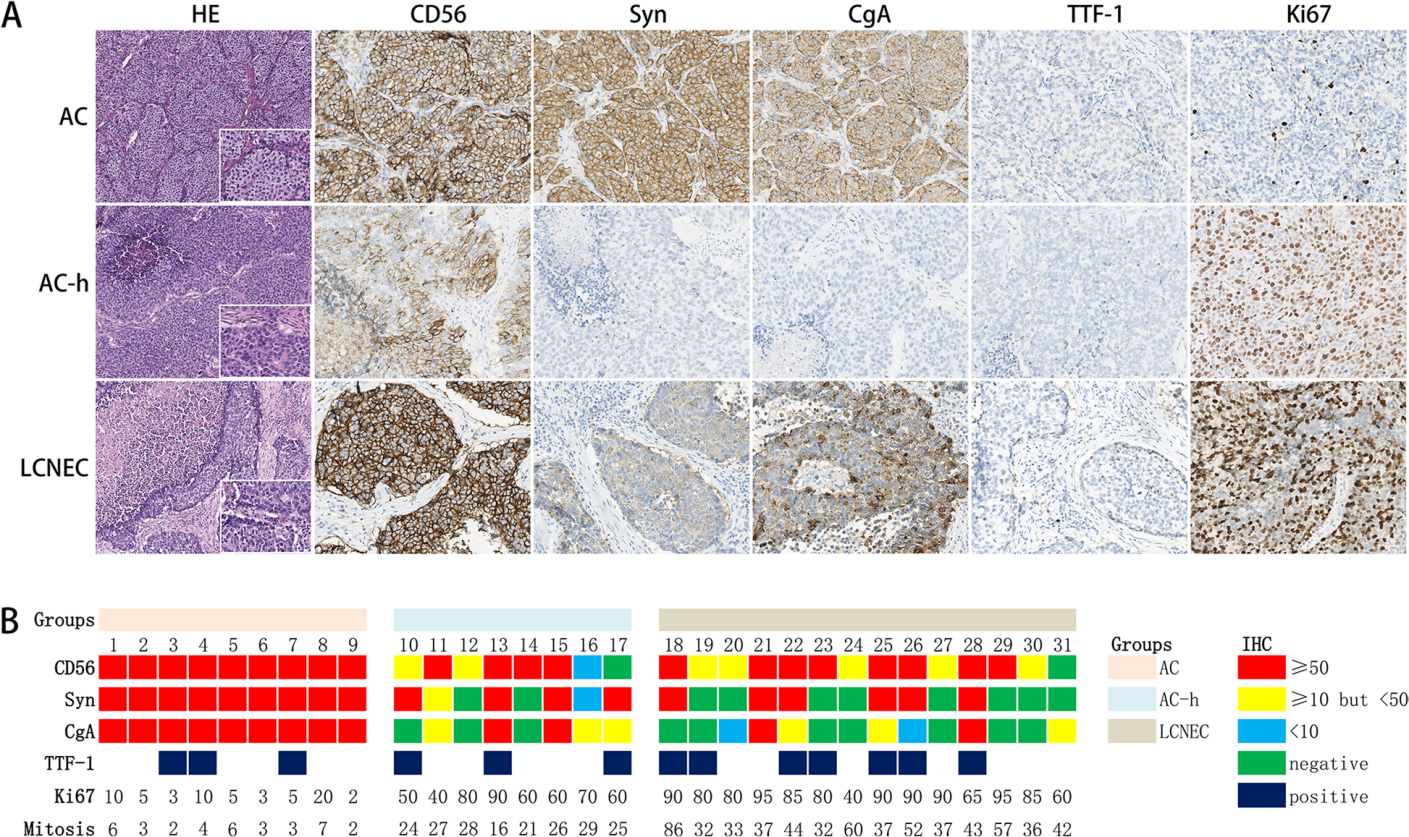
**A** Representative HE and IHC imagines of AC, AC-h and LCNEC under light microscope at × 100 magnification (inset × 400) for HE and at × 200 magnification for IHC; **B** IHC and mitosis results of the all 31 patients. Case: case number; Ki67: calculated on the hot spot area under the field of view × 400; Mitosis: counted on the 5^th^ edition WHO diagnostic criteria and for these samples which the mitoses near the threshold of two or ten per 2mm^2^, the average of counts in at least three hot sets of per 2mm^2^ were token as the result

Histopathological analysis of AC-h revealed classical features of NET (tumour cells were relatively uniform, featuring moderate to abundant cytoplasm and finely nuclear chromatin) and NEC (focal necrosis, and even extensive necrosis in four cases) (Fig. 2). IHC for neuroendocrine (NE) markers (CD56, Syn, and CgA), TTF-1 and Ki67 was performed on 31 samples. The most sensitive neuroendocrine marker was CD56 (93.5%), followed by Syn and CgA (both were 67.7%). All ACs exhibited strong positivity for the three NE markers, while the expression mode was more variable in AC-hs and LCNECs. After reviewing all the slices, mitosis in AC-hs (average, 25 per 2 mm^2^) was found to differ significantly from that of ACs (average, 4 per 2 mm^2^) and LCNEC (average, 45 per 2 mm^2^) (*P* < 0.001). Similar results could be observed using Ki67 (*P* < 0.001).

### Genomic features

**Fig. 3:**
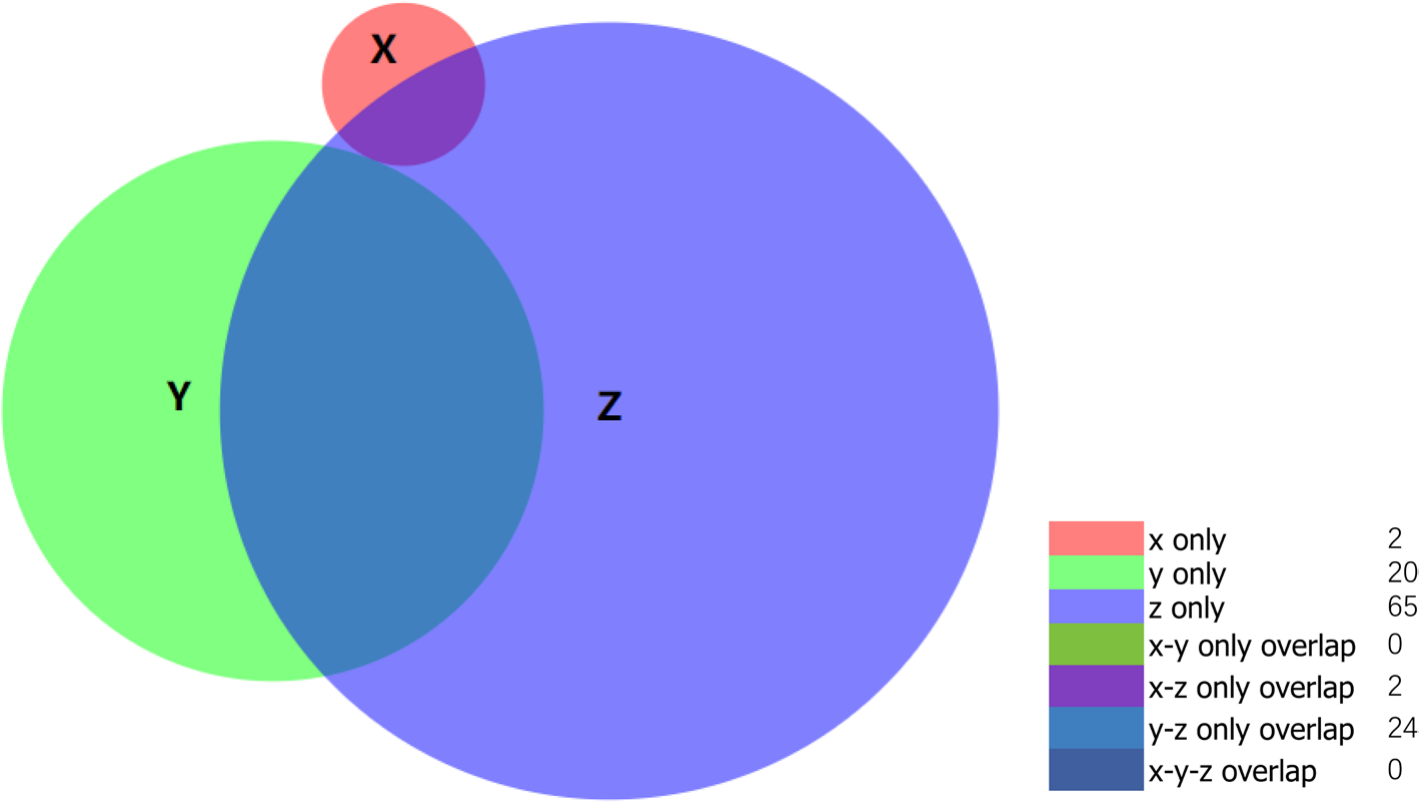
The 425-exon sequencing in 28 pure primary tumour samples revealing 113 altered genes. Abbreviations: X: AC; Y: AC-h; Z: LCNEC; x only: *AKT1, PDK1;* y only: *CREBBP, SMARCA4, NKX2-1, KMT2B, IKBKE, TEK, PIK3R1, RET, SETBP1, EPHA3, PRDM1, TERC, AKT2, TOP2A, CCNE1, CBL, NOTCH2, IDH1, CTCF, AXL*; z only: *RICTOR, MCL1, ATRX, MYC, ABCB1, FAT1, PALB2, FLT4, TERT, ARID1A, EPHA2, NTRK3, NOTCH1, PDGFRA, BRAF, JAK1, EPHA5, DAXX, ZNF217, ERBB4, TSC2, GATA2, PPARD, SDHC, SDHA, FLCN, TUBB4A, PALLD, SRC, SMAD3, EZH2, BARD1, ATM, AKT3, TPMT, GRIN2A, MAP2K2, BTK, GATA4, MET, RUNX1, BIRC3, CHEK1, DENND1A, PTK2, AXIN2, TGFBR2, PMS2, ARID1B, EP300, POLH, DUSP2, MYCN, IGF1R, DPYD, PREX2, CYSLTR2, CHEK2, EGFR, DDR2, RAD54L, WRN, LZTR1, LHCGR, BRCA1*; x–z only overlap: *ROS1, MEN1*; y–z only overlap: *TP53, PTEN, KEAP1, APC, RB1, PKHD1, NTRK1, KIT, PIK3C3, PIK3CA, SOX2, GRM3, LRP1B, GNAS, KRAS, IL7R, POLE, NF1, CRKL, VEGFA, DLL3, CDKN2A, JAK3, STK11*

**Fig. 4:**
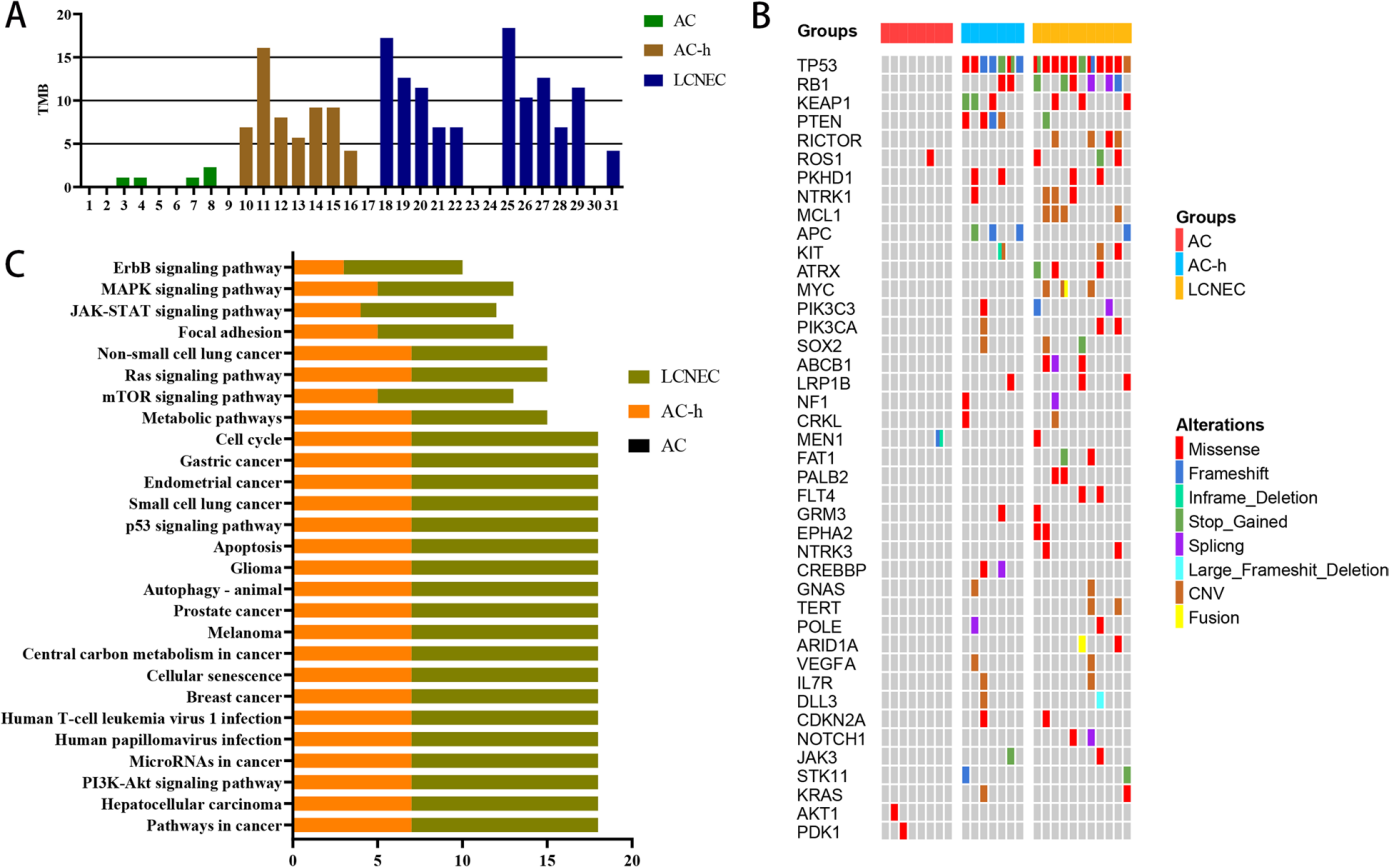
Abbreviations: (**A**) *TMB* Tumour mutation burden (mutations / MB); Case 1 and 17: do not acquire sufficient sequencing depth due to DNA degradation; Case 5, 6 and 9: do not detect genetic mutations; Case 23, 24 and 30: except owing to diagnosed as combined LCNEC; (**B**) The 42 genes consisted by the selected 26 tumour samples (due to DNA degradation, case 1 and 17 did not acquire a sufficient sequencing depth, meanwhile, for case 2, 5, 6 and 9 we did not detect any genetic mutations) involved: 40 genes which occurred more than one time and 2 genes which the atypical carcinoid samples involved; (**C**)The abscissa represents the number of cases involved in; The ordinate represents the involved mutation paths; The pathways which involved at least there of the top ten genes: Pathways in cancer; Hepatocellular carcinoma; PI3K-Akt signaling pathway; MicroRNAs in cancer; Human papillomavirus infection; Human T-cell leukemia virus 1 infection; Breast cancer; Cellular senescence; Central carbon metabolism in cancer; Melanoma; Prostate cancer; Autophagy-animal; Glioma; Apoptosis; p53 signaling pathway; Small cell lung cancer; mTOR signaling pathway; Metabolic pathways; Endometrial cancer; Gastric cancer and Cell cycle; The other 6 paths reported usually been seen in LCNEC: Ras signaling pathway, Non-small cell lung cancer, Focal adhesion, JAK-STAT signaling pathway, MAPK signaling pathway, ErbB signaling pathway

The 425-exon sequencing was performed by unsupervised clustering in 28 pure primary tumour samples (Nanjing Geneseeq Technology Inc.), revealing 113 altered genes (Fig. 3). Tumour mutational burden (TMB) is defined as the number of somatic cells, coding number, base subsets, and index of each detected genome. Our analysis revealed that the TMB value in ACs (average, 0.7 mutations/MB) contrasted with those of AC-hs (average, 8.5 mutations/MB) and LCNECs (average, 10.8 mutations/MB) (*P* < 0.001) (Fig. 4A).

At the single gene level, among the commonly mutated genes, differences in *P*-values were found between these three groups. The most commonly mutated genes were *TP53* (*P* < 0.001) and *RB1* (*P* = 0.039), with a significant bi-alteration rate (*P* = 0.039), followed by *PTEN* (*P* = 0.011), *RICTOR* and *MCL1* (*P* = 0.040), APC (*P* = 0.054), *KEAP1* (*P* = 0.132), *NTRK1* (*P* = 0.265), *ROS1* (*P* = 0.284) and *PKHD1* (*P* = 0.293) (Fig. 4B). Furthermore, we matched the gene mutation sites with the corresponding protein locations of the top ten genes, which showed that case 10 and 20 had the same mutation in *TP53* (824G > T), corresponding to the protein location C275F. Moreover, several highly similar mutated sites existed between the AC-h and LCNEC groups (Table S1).

The KEGG web was used to search the pathways in which the top ten mutated genes are involved. As a result, the pathways which involved at least three of the top ten genes (*n* = 21) were collected (Table S2), within the six other common mutation pathways in LCNEC listed in Fig. 4C [20–23].

## Discussion

Lung neuroendocrine tumours represent a group of heterogeneous malignancies, and according to the 5^th^ edition of WHO, apparent differences exist between NET and NEC for NETs lack mutations in *TP53, RB1, KRAS*, and *STK11/KEAP1,* while, in 40% of cases they have mutations in chromatin-remodelling genes [13]. Genetic screening of 45 surgically resected pure-LCNEC by Rekhtman et al. found two cases of carcinoid-like molecular profiles. Moreover, these two cases displayed apparent carcinoid-like morphology, although the elevated proliferation rate above the cut-off value accepted of NET had led them to be classified as LCNEC [17]. Similar results were found in other studies [8, 24]. This type of neoplasm in the pancreas is classified as NET G3, which has a common mutation lineage with NET G1 and NET G2 and can evolve from G1/G2, and has nothing to do with the progress of NEC [15]. Although some researchers believe these tumours generally correspond to those regarded as NETs in the pancreas, however, in the lung, the WHO still classifies these neoplasms as LCNEC, although their prognosis has been suggested to be different from traditional LCNECs, which means more clinical, pathological, and genetic studies are needed to determine how to fit these rare tumours into the classification [1, 13]. Thus, it is essential to recognise these grey-zone AC-hs to improve disease classification and avoid incorrect clinical treatment choices.

Previous studies have pointed out that AC occurred in younger patients than LCNEC [15, 25–27], and comparison with the age is mentioned by WHO, the patients with AC or LCNEC in this study tended to be younger [13]. Unlike AC, which does not show a strong association with cigarette smoking [6, 15, 25, 28, 29], and is slightly more common in women [9, 11, 26, 27], AC-h primarily affects middle-aged men with a smoking history. Caplin ME et al. reported that well-differentiated lung NETs are usually located centrally in the main or lobar bronchi (up to 80% of tumours) [30–32], although some reports have opposed this view [4, 27, 33]. Results indicated all three groups had a trend of occurring in the periphery.

The WHO (4th edition) diagnostic criteria recommends the use of NE markers to confirm a diagnosis of neuroendocrine differentiation [1] the guidelines for the diagnosis and management of pNEN (2020) support this point [34], and this is reiterated in the 5th edition [13]. Our results suggest that compared with HE, IHC is more accurate in diagnosing lung carcinoids, in particular, Syn and CgA can be used to distinguish NET from NEC [35]. TTF-1, a putative regulator of neurogenesis expressed in pNEC at various sites [36, 37] was found to be mostly positive in peripheral NETs. However, for LCNEC, the positive expression rate of TTF-1 (50%) was slightly lower than that described by WHO (70%) [13].

The Ki67 antigen can identify proliferating cells and is important for distinguishing NETs and NECs, especially in small squeezed biopsy samples [38], and the value was now increased to 30% for AC [13]. Based on this change, some studies have used Ki67 to identify the proper cut-off value of these four pNENs, however, there was no conclusive result even in resected samples [6, 10, 38–40]. As shown in Fig. 2, malignant divergences existed in both the proliferation level and mitotic counts in these three neoplasms.

Global genomic studies have demonstrated that AC has a low mutation rate (0.3–0.4 mutations/Mb) [29, 41] and very few genetic changes [42]. High-frequency mutations include *KIT*, *ERBB4,* and *MET* [35, 43]. Unlike NECs, mutations in chromatin-remodelling genes are observed in approximately 40–50% of NET cases [3, 10, 41, 44]. For example, *MEN1* (11–22%) is the most frequently mutated gene with somatic mutations in lung carcinoids [2, 41, 45]. Other statistically significant commonly mutated genes include *EIF1AX* and *ARID1A* [41]. However, except for the *MEN1* mutation in case 8, NGS testing indicated no other commonly mutated genes in AC. Conversely, three other genes were found to be mutated in this cohort—*ROS1* (a common driver gene), *PDK1,* and *AKT1*. These have never previously been reported for AC and should be investigated further.

Sazonova et al. recently applied IHC to surgical samples from 18 lung cancer patients, four of whom had defined borderline tumours (LCNEC with a low mitotic count and carcinoid-like morphology). They found that all AC and borderline tumours had preserved P53/RB expression [8]. Indeed, Meder et al. and Nakamura et al. reported that *TP53* and *RB1* gene inactivation are among the hallmarks of SCLC, existing in approximately 39.3% of SCLC cases. At the same time, for LCNEC, the rate was approximately 36.8% [41, 46]. Biallelic alterations of *TP53* and *RB1* are strikingly correlated with high-grade NECs, although uncommon in pNETs [2, 3, 21, 47]. The co-mutation of *TP53*/*RB1*, and common mutations in LCNECs like *TP53, RB1, MEN1, STK11, KEAP1,* and *KRAS* were commonly seen in AC-hs in our study. Furthermore, LCNEC is a heterogeneous tumour, which can be divided into three genotypes: (i) an SCLC-like subtype with biallelic inactivation of *TP53 and RB1*, (ii) a non-small-cell-like subtype with mutations in *TP53* and *STK11/KEAP1*, (iii) and a carcinoid-like subtype sharing the low TMB and *MEN1* changes seen in lung carcinoids [2, 13, 17, 18, 21, 37, 48, 49]. These results, taken together, suggests a way to classify the AC-hs effectively. The extensive TMB fluctuation range of AC-hs also supports this. In addition, according to the summary results of the three neoplasms gene mutations provided in Fig. 3, there is no shared mutation gene type between AC-h and AC. This result reiterated that according to our data, in the lung, there exist a big difference between AC-h and AC, which is completely different from the existing research of NET G3 in pancreatic NENs.

Subsequently, the 27 pathways considered in this study showed a high degree of similarity in the level of involvement of AC-h and LCNEC, suggesting that differences do exist between these tumours and ACs.

Tumorigenesis results from multiple factors, and abnormal activation of the PI3K-Akt-mTOR pathway is a frequent event in the non-small cell lung cancer development [20, 22, 23, 50]. The mammalian target of rapamycin (mTOR) serves as a signal amplifier in this pathway [22, 51]. It is generally believed that mutated genes in the PI3K/AKT/mTOR pathway are significantly related to the occurrence of NEC [2, 3, 17, 52]. However, inconsistent findings have been reported in pNEN, with some reporting that most mutated genes in NETs are located in this pathway [53] or that these mutated genes exhibit a high degree of participation [3, 52]. Our data support the conclusion that the mutated genes in AC are involved in these pathways. In addition, alterations in this pathway were far more common in LCNEC patients than previously reported [3, 17, 54].

Regarding survival and prognosis, several studies have suggested a similar prognosis between LCNEC and SCLC [55–57], which is significantly poorer than that of AC [55, 58]. The five-year survival rate of LCNEC patients is 15–57% [25, 59–63], while that of AC patients is 44–87% [6, 26, 59–61]. We attempted to enrol as many cases as possible but limited by the rare incidence and short DNA storage period, significantly different OS outcomes among these three tumour types were not obtained (*P* = 0.123). However, a trend from the available information suggested that AC-hs seemed to have a better prognosis than LCNECs.

## Conclusion

We present basic information on the clinical features and genomic changes in 31 tumour samples. Despite limitations in the number of cases and the lack of effective differential data for OS, we can still clearly see that AC-h and LCNEC patients are more similar to each other with respect to demographic characteristics, tumour size and location, clinical presentation, pathological data, and genomic changes. Thus, we believe that carcinoid morphology with increased mitotic index is more similar to LCNEC, but has a better survival prognosis. However, to test this hypothesis, a larger cohort study is needed and until these neoplasms are better classified, we endorse that it is necessary to add a diagnostic note stating the histological morphology, mitotic count and Ki67 index of this type of tumour in the clinical diagnosis process.

## Supplementary Information

**Additional file 1.** Gene mutation sites and affected protein changes.

**Additional file 2.** The pathways in which the top ten mutated genes are involved.

## Data Availability

The datasets used and analysed during the current study are available from the corresponding author on reasonable request.The KEGG datasets were obtained from:- Kanehisa, M. and Goto, S.; KEGG: Kyoto Encyclopedia of Genes and Genomes. Nucleic Acids Res. 28, 27–30 (2000). [PMID:10592173]- Kanehisa, M; Toward understanding the origin and evolution of cellular organisms. Protein Sci. 28, 1947–1951 (2019). [PMID:31441146]- Kanehisa, M., Furumichi, M., Sato, Y., Ishiguro-Watanabe, M., and Tanabe, M.; KEGG: integrating viruses and cellular organisms. Nucleic Acids Res. 49, D545-D551 (2021). [PMID:33125081].

## Abbreviations

WHO: World health organization
LCNEC: Large cell neuroendocrine carcinoma
pNENs: Pulmonary neuroendocrine neoplasms
NENs: Neuroendocrine neoplasms
NETs: Neuroendocrine tumours
NECs: Neuroendocrine carcinomas
TC: Typical carcinoid
AC: Atypical carcinoid
SCLC: Small cell (lung) carcinoma
AC-h: PNENs have an AC-like morphology with elevated mitotic counts over 10 per 2 mm^2^
OS: Overall survival
Syn: Synaptophysin
CgA: Chromogranin A
IHC: Immunohistochemica
KEGG web: The Kyoto Encyclopedia of Genes and Genomes website
NE: Neuroendocrine
TMB: Tumour mutational burden
