# Supplementary material for: Clinic and genetic similarity assessments of atypical carcinoid, neuroendocrine neoplasm with atypical carcinoid morphology and elevated mitotic count and large cell neuroendocrine carcinoma
Source: BMC Cancer. 2022 Mar 24;22:321. doi: 10.1186/s12885-022-09391-w (PMC8951721; doi:10.1186/s12885-022-09391-w)
Supplement: Supplementary file 1 — Additional file 1. Gene mutation sites and affected protein changes. [file 12885_2022_9391_MOESM1_ESM.pdf]

Supplement 1: Gene mutation sites and affected protein changes

Mutant gene	Cases	Gene mutation point	Protein mutation point	Spacer codon
	10<br>20	c.824G>T	p.C275F	
	18<br>21	c.746G>T	p.R249M	
	10<br>11	c.672G>T<br>c.480_481delGGinsTT	p.E224D<br>p.M160_A161delinsIS	64
	10<br>12	c.824G>T<br>c.455delC	p.C275F<br>p.P152RfsX18	123
	10<br>18	c.824G>T<br>c.746G>T	p.C275F<br>p.R249M	26
	10<br>19	c.824G>T<br>c.374_375delCGinsAT	p.C275F<br>p.T125N	150
	10<br>28	c.672G>T<br>c.396G>C	p.E224D<br>p.K132N	92
	11<br>28	c.480_481delGGinsTT<br>c.396G>C	p.M160_A161delinsIS<br>p.K132N	28
	12<br>18	c.455delC<br>c.746G>T	p.P152RfsX18<br>p.R249M	97
	12<br>19	c.455delC<br>c.374_375delCGinsAT	p.P152RfsX18<br>p.T125N	27
	12<br>27	c.455delC<br>c.428T>A	p.P152RfsX18<br>p.V143E	9
	12<br>29	c.455delC<br>c.833C>G	p.P152RfsX18<br>p.P278R	126
	13<br>14	c.277delC<br>c.1015G>T	p.L93Cfs*30<br>p.E339*	246
	13<br>15	c.277delC<br>c.817C>T	p.L93Cfs*30<br>p.R273	180
	13<br>15	c.277delC<br>c.892G>T	p.L93Cfs*30<br>p.E298*	205
	13<br>18	c.277delC<br>c.538G>T	p.L93Cfs*30<br>p.E180X	87
	13<br>22	c.277delC<br>c.730G>T	p.L93Cfs*30<br>p.G244C	151
	13<br>25	c.277delC<br>c.202G>T	p.L93Cfs*30<br>p.E68X	25
	13<br>26	c.277delC<br>c.217G>T	p.L93Cfs*30<br>p.V73L	20
	14<br>15	c.1015G>T<br>c.817C>T	p.E339*<br>p.R273	66
	14	c.1015G>T	p.E339*	41

	15	c.892G>T	p.E298*	41
	14<br>18	c.1015G>T<br>c.538G>T	p.E339*<br>p.E180X	159
	14<br>22	c.1015G>T<br>c.730G>T	p.E339*<br>p.G244C	95
TP53	14<br>25	c.1015G>T<br>c.202G>T	p.E339*<br>p.E68X	271
	14<br>26	c.1015G>T<br>c.217G>T	p.E339*<br>p.V73L	266
	15	c.817C>T<br>c.892G>T	p.R273<br>p.E298*	25
	15<br>18	c.817C>T<br>c.538G>T	p.R273<br>p.E180X	93
	15<br>18	c.892G>T<br>c.538G>T	p.E298*<br>p.E180X	118
	15<br>22	c.817C>T<br>c.730G>T	p.R273<br>p.G244C	29
	15<br>22	c.892G>T<br>c.730G>T	p.E298*<br>p.G244C	54
	15<br>25	c.817C>T<br>c.202G>T	p.R273<br>p.E68X	205
	15<br>25	c.892G>T<br>c.202G>T	p.E298*<br>p.E68X	230
	15<br>26	c.817C>T<br>c.217G>T	p.R273<br>p.V73L	200
	15<br>26	c.892G>T<br>c.217G>T	p.E298*<br>p.V73L	225
	18<br>22	c.538G>T<br>c.730G>T	p.E180X<br>p.G244C	64
	18<br>25	c.538G>T<br>c.202G>T	p.E180X<br>p.E68X	112
	18<br>26	c.538G>T<br>c.217G>T	p.E180X<br>p.V73L	107
	18<br>29	c.746G>T<br>c.833C>G	p.R249M<br>p.P278R	29
	19<br>21	c.374_375delCGinsAT<br>c.746G>T	p.T125N<br>p.R249M	124
	19<br>27	c.374_375delCGinsAT<br>c.428T>A	p.T125N<br>p.V143E	18
	19<br>29	c.374_375delCGinsAT<br>c.833C>G	p.T125N<br>p.P278R	153
	20<br>29	c.824G>T<br>c.833C>G	p.C275F<br>p.P278R	3
	21<br>27	c.746G>T<br>c.428T>A	p.R249M<br>p.V143E	106

	22<br>25	c.730G>T<br>c.202G>T	p.G244C<br>p.E68X	176
	22<br>26	c.730G>T<br>c.217G>T	p.G244C<br>p.V73L	171
	25<br>26	c.202G>T<br>c.217G>T	p.E68X<br>p.V73L	5
	27<br>29	c.428T>A<br>c.833C>G	p.V143E<br>p.P278R	135

Mutant gene	Cases	Gene mutation point	Protein mutation point	Spacer codon
RB1	15<br>18	c.2134T>C<br>c.610G>T	p.C712R<br>p.E204X	508
	15<br>21	c.2134T>C<br>c.1363C>T	p.C712R<br>p.R455X	257
	15<br>22	c.2134T>C<br>c.1981C>T	p.C712R<br>p.R661W	51
	18<br>22	c.610G>T<br>c.1981C>T	p.E204X<br>p.R661W	457
	21<br>22	c.1363C>T<br>c.1981C>T	p.R455X<br>p.R661W	206

Mutant gene	Cases	Gene mutation point	Protein mutation point	Spacer codon
PTEN	10<br>19	c.601G>A<br>c.565A>T	p.E201K<br>p.R189X	12

Mutant gene	Cases	Gene mutation point	Protein mutation point	Spacer codon
KEAP1	10<br>11	c.1687C>T<br>c.1027G>T	p.Q563*<br>p.E343X	220
	10<br>20	c.1687C>T<br>c.484T>A	p.Q563*<br>p.Y162N	401
	10<br>25	c.1687C>T<br>c.1237C>A	p.Q563*<br>p.R413S	150
	10<br>31	c.1687C>T<br>c.997G>T	p.Q563*<br>p.G333C	230
	11<br>31	c.1027G>T<br>c.997G>T	p.E343X<br>p.G333C	10
	11<br>20	c.1027G>T<br>c.484T>A	p.E343X<br>p.Y162N	181
	11<br>25	c.1027G>T<br>c.1237C>A	p.E343X<br>p.R413S	70
	20<br>25	c.484T>A<br>c.1237C>A	p.Y162N<br>p.R413S	251
	20<br>25	c.484T>A<br>c.1237C>A	p.Y162N<br>p.R413S	251
	25<br>31	c.1237C>A<br>c.997G>T	p.R413S<br>p.G333C	80

Mutant gene	Cases	Gene mutation point	Protein mutation point	Spacer codon

ROS1	18<br>27	c.693G>T<br>c.5433C>A	p.R231S<br>p.C1811X	1580

Mutant gene	Cases	Gene mutation point	Protein mutation point	Spacer codon
PKHD1	11	c.571T>A<br>c.8659G>T	p.C191S<br>p.A2887S	2696
	11<br>14	c.571T>A<br>c.11101G>A	p.C191S<br>p.V3701I	3510
	11<br>14	c.8659G>T<br>c.11101G>A	p.A2887S<br>p.V3701I	814
	11<br>27	c.8659G>T<br>c.10660G>A	p.A2887S<br>p.E3554K	667
	11<br>27	c.8030C>A<br>c.10142C>G	p.P2677Q<br>p.S3381C	704
	11<br>27	c.571T>A<br>c.7003A>G	p.C191S<br>p.R2335G	2144
	11<br>27	c.8659G>T<br>c.7003A>G	p.A2887S<br>p.R2335G	552
	11<br>27	c.571T>A<br>c.10660G>A	p.C191S<br>p.E3554K	3363
	14<br>27	c.11101G>A<br>c.7003A>G	p.V3701I<br>p.R2335G	1366
	14<br>27	c.11101G>A<br>c.10660G>A	p.V3701I<br>p.E3554K	147
	27	c.7003A>G<br>c.10660G>A	p.R2335G<br>p.E3554K	1219

Abberation: Cases: cases of patients; Protein mutation point: the corresponding protein locations
