# Supplementary material for: Clinic and genetic similarity assessments of atypical carcinoid, neuroendocrine neoplasm with atypical carcinoid morphology and elevated mitotic count and large cell neuroendocrine carcinoma
Source: BMC Cancer. 2022 Mar 24;22:321. doi: 10.1186/s12885-022-09391-w (PMC8951721; doi:10.1186/s12885-022-09391-w)
Supplement: Supplementary file 2 — Additional file 2. The pathways in which the top ten mutated genes are involved. [file 12885_2022_9391_MOESM2_ESM.pdf]

Supplement 2: The pathways in which the top ten mutated genes are involved

**Pathway**	TP53	RB1	RICTOR	ROS1	NTRK1	PTEN	KEAP1	MCL1	APC	**Total**
Pathways in cancer	+	+			+	+	+		+	**6**
Hepatocellular carcinoma	+	+				+	+		+	**5**
PI3K-Akt signaling pathway	+				+	+		+		**4**
MicroRNAs in cancer	+					+		+	+	**4**
Human papillomavirus infection	+	+				+			+	**4**
Human T-cell leukemia virus 1 infection	+	+				+			+	**4**
Breast cancer	+	+				+			+	**4**
Cellular senescence	+	+				+				**3**
mTOR signaling pathway		+	+			+				**3**
Central carbon metabolism in cancer	+				+	+				**3**
Melanoma	+	+				+				**3**
Prostate cancer	+	+				+				**3**
Autophagy - animal	+	+				+				**3**
Glioma	+	+				+				**3**
Apoptosis	+				+			+		**3**
p53 signaling pathway	+	+				+				**3**
Small cell lung cancer	+	+				+				**3**
Gastric cancer	+	+							+	**3**
Metabolic pathways		+				+			+	**3**
Endometrial cancer	+					+			+	**3**
Cell cycle	+	+							+	**3**
MAPK signaling pathway	+				+					**2**
Human cytomegalovirus infection	+	+								**2**
Hepatitis B	+	+								**2**
Endocrine resistance	+	+								**2**
Chronic myeloid leukemia	+	+								**2**
Viral carcinogenesis	+	+								**2**
Kaposi sarcoma-associated herpesvirus infection	+	+								**2**
Non-small cell lung cancer	+	+								**2**
JAK-STAT signaling pathway		+						+		**2**
Pancreatic cancer	+	+								**2**
Bladder cancer	+	+								**2**
Hepatitis C	+	+								**2**
Transcriptional misregulation in cancer	+				+					**2**

Longevity regulating pathway	+	+								**2**
Biosynthesis of secondary metabolites		+				+				**2**
Epstein-Barr virus infection	+	+								**2**
Neurotrophin signaling pathway	+				+					**2**
Fluid shear stress and atherosclerosis	+						+			**2**
Sphingolipid signaling pathway	+					+				**2**
Huntington disease	+	+								**2**
Thyroid cancer	+				+					**2**
Inflammatory mediator regulation of TRP channels		+			+					**2**
Complement and coagulation cascades		+		+						**2**
Alzheimer disease		+							+	**2**
Colorectal cancer	+								+	**2**
Cushing syndrome		+							+	**2**
Regulation of actin cytoskeleton		+							+	**2**
Hippo signaling pathway	+								+	**2**
Wnt signaling pathway	+								+	**2**
Th17 cell differentiation		+							+	**2**
Microbial metabolism in diverse		+							+	**2**
Th1 and Th2 cell differentiation		+							+	**2**
Quorum sensing		+							+	**2**
Inflammatory bowel disease (IBD)		+							+	**2**
Basal cell carcinoma	+								+	**2**
Ras signaling pathway					+					**1**
EGFR tyrosine kinase inhibitor resistance						+				**1**
FoxO signaling pathway						+				**1**
Proteoglycans in cancer	+									**1**
Thyroid hormone signaling pathway	+									**1**
Focal adhesion						+				**1**
Shigellosis	+									**1**
Calcium signaling pathway		+								**1**
cAMP signaling pathway		+								**1**
Gap junction		+								**1**
Herpes simplex virus 1 infection	+									**1**
MAPK signaling pathway - fly				+						**1**
Measles	+									**1**
PD-L1 expression and PD-1 checkpoint pathway in cancer						+				**1**
Parkinson disease	+									**1**

Axon regeneration						+				1
Platinum drug resistance	+									1
Neuroactive ligand-receptor interaction		+								1
Autophagy - yeast		+								1
Two-component system		+								1
Vascular smooth muscle contraction			+							1
Bile secretion		+								1
Spinocerebellar ataxia		+								1
GnRH secretion		+								1
Epithelial cell signaling in Helicobacter pylori infection		+								1
Insulin resistance						+				1
Cytokine-cytokine receptor interaction		+								1
Ovarian steroidogenesis		+								1
Inositol phosphate metabolism						+				1
Regulation of lipolysis in adipocytes		+								1
Carbon metabolism		+								1
Longevity regulating pathway - worm						+				1
cGMP-PKG signaling pathway		+								1
Aldosterone synthesis and secretion		+								1
Cortisol synthesis and secretion		+								1
Amyotrophic lateral sclerosis (ALS)	+									1
Phosphatidylinositol signaling system						+				1
Cholesterol metabolism		+								1
NOD-like receptor signaling pathway	+									1
Vitamin digestion and absorption		+								1
Mitophagy - animal	+									1
Fat digestion and absorption		+								1
Phagosome		+								1
Adrenergic signaling in cardiomyocytes		+								1
Streptomycin biosynthesis		+								1
Renin secretion		+								1
Salivary secretion		+								1
Pancreatic secretion		+								1
Dilated cardiomyopathy (DCM)		+								1
Biosynthesis of phenylpropanoids						+				1
Protein digestion and absorption		+								1
Ferroptosis	+									1

Methane metabolism		+								**1**
Biosynthesis of terpenoids and steroids						+				**1**
Nonribosomal peptide structures		+								**1**
Bacterial secretion system		+								**1**
Sesquiterpenoid and triterpenoid						+				**1**

Abberation: Pathway: the name of the pathway; Total: the total number of the genes involved in this pathway; +: this gene is involved in this pathway.
